# (1*S*,4*S*,5*S*,8*R*)-8-Nitro­oxy-2,6-dioxa­bicyclo­[3.3.0]octan-4-yl 3,4,5-triacetoxy­benzoate

**DOI:** 10.1107/S1600536809042524

**Published:** 2009-10-23

**Authors:** Yue Zhang, Ping-An Wang, Meng-Yao Zhang, Xiao-Li Sun

**Affiliations:** a Department of Chemistry, School of Pharmacy, Fourth Military Medical University, Changle West Road 17, 710032, Xi-An, People’s Republic of China

## Abstract

In the title compound, C_19_H_19_NO_13_, one of the two fused furan­ose rings adopts an envelope conformation whereas the other displays a twisted conformation. The crystal structure is stabilized by inter­molecular C—H⋯π inter­actions between a methine H atom and the triacetoxy­phenyl ring of an adjacent mol­ecule, and by weak non-classical inter­molecular C—H⋯O hydrogen bonds.

## Related literature

For the preparation of the title compound, see: Velazquez *et al.* (2007 ▶), Calmès *et al.*(2003 ▶). For related structures, see: Ezhilmuthu *et al.* (2008 ▶). For the bioactivity of the title compound, see: Rigas & Williams (2008 ▶); Carini *et al.* (2002 ▶). For puckering parameters, see: Cremer & Pople (1975 ▶); Rao *et al.* (1981 ▶).  For the determination of the absolute structure, see: van Koningsveld *et al.* (1984 ▶); Brown *et al.* (2000 ▶).
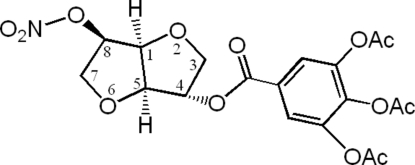


## Experimental

### 

#### Crystal data

C_19_H_19_NO_13_
*M* *_r_* = 469.35
Monoclinic, 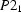

*a* = 10.8053 (19) Å
*b* = 6.5941 (12) Å
*c* = 16.075 (3) Å
β = 108.243 (3)°
*V* = 1087.8 (3) Å^3^
*Z* = 2
Mo *K*α radiation
μ = 0.12 mm^−1^
*T* = 296 K
0.31 × 0.25 × 0.14 mm

#### Data collection

Bruker APEXII CCD area-detector diffractometer
Absorption correction: multi-scan (*SADABS*; Bruker, 2000 ▶) *T*_min_ = 0.962, *T*_max_ = 0.983
5474 measured reflections
2112 independent reflections
1394 reflections with *I* > 2σ(*I*)
*R*_int_ = 0.023

#### Refinement

*R*[*F* ^2^ > 2σ(*F* ^2^)] = 0.039
*wR*(*F* ^2^) = 0.105
*S* = 1.02
2112 reflections
301 parameters
H-atom parameters constrained
Δρ_max_ = 0.12 e Å^−3^
Δρ_min_ = −0.14 e Å^−3^

### 

Data collection: *APEX2* (Bruker, 2000 ▶); cell refinement: *SAINT* (Bruker, 2000 ▶); data reduction: *SAINT*; program(s) used to solve structure: *SHELXS97* (Sheldrick, 2008 ▶); program(s) used to refine structure: *SHELXL97* (Sheldrick, 2008 ▶); molecular graphics: *ORTEPIII* (Burnett & Johnson, 1996 ▶) and *ORTEP-3 for Windows* (Farrugia, 1997 ▶); software used to prepare material for publication: *SHELXTL* (Sheldrick, 2008 ▶) and *PLATON* (Spek, 2009 ▶).

## Supplementary Material

Crystal structure: contains datablocks I, global. DOI: 10.1107/S1600536809042524/dn2497sup1.cif

Structure factors: contains datablocks I. DOI: 10.1107/S1600536809042524/dn2497Isup2.hkl

Additional supplementary materials:  crystallographic information; 3D view; checkCIF report

## Figures and Tables

**Table 1:** Hydrogen-bond geometry (Å, °)

*D*—H⋯*A*	*D*—H	H⋯*A*	*D*⋯*A*	*D*—H⋯*A*
C5—H5⋯*Cg*1^i^	0.98	2.56	3.365 (13)	139
C4—H4⋯O42^ii^	0.98	2.53	3.507 (15)	177
C43—H43⋯O11^i^	0.93	2.51	3.25 (3)	136
C47—H47⋯O42^iii^	0.93	2.52	3.314 (14)	144
C11—H11*B*⋯O83^iv^	0.96	2.52	3.39 (3)	151

Symmetry codes: (i) 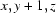; (ii) 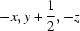; (iii) 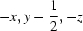; (iv) 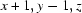. *Cg1* is the centroid of the C42–C47 phenyl ring.

**Fig. 1.**
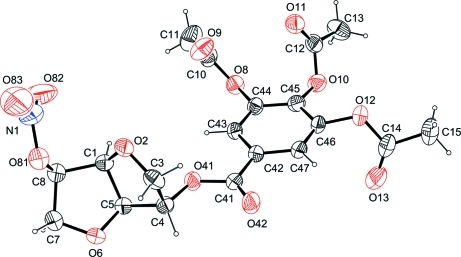
Molecular structure of the title compound (I), showing the atom labeling scheme. Displacement ellipsoids are drawn at the 30% probability level. H atoms are represented as small spheres of arbitrary radii.

## supplementary crystallographic information

### Comment

The title compound is synthesized by esterification of 3,4,5-triacetoxybenzoic acid with Isosorbide Mononitrate.It can be rapidly metabolized to 3,4,5-trihydroxybenzoic acid and Isosorbide Mononitrate *in vivo*(Carini *et al.*,2002). 3,4,5-trihydroxybenzoic acid is a bioactivesubstance which can scavenge oxygen free radicals and Isosorbide Mononitrate is a classical nitric oxide-donor drug. This bifunctional molecule may have better bioactivity but do bring fewer side effect.

The molecule is built up from the isosorbide mononitrate skeleton substituted on C4 by the 3,4,5-triacetoxybenzoate (Fig. 1). The two fused furanose rings have slightly different conformation. Indeed, the Cremer & Pople (1975) puckering parameters for the C1-O2-C3-C4-C5 ring are Q(2) = 0.359 (4)Å and φ(2) = 289.1 (6)° whereas those for the C5-O6-C7-C8-C1 ring are Q(2) = 0.334 (4)Å and φ(2) = 343.4 (7)°. These values indicate that these two rings have different extent of puckering caused by the different substituent group in the ring. The pseudorotation parameters (Rao *et al.* 1981) for C1-O2-C3-C4-C5 ring are P = 20.8 (4)° & τ(*M*) = 37.3 (2)° for the C5—C4 reference bond with the closest pucker descriptor being enveloped on C(4) and those for C5-O6-C7-C8-C1 ring are P = 75.4 (4)° and τ(*M*) = 35.7 (3)° for the C8—C1 reference bond with the closest puckering descriptor being twisted on C5—O6.

Owing to the know absolute configuration of the starting isosorbide mononitrate, the absolute configuration of the title compound could be deduced to be 1S,4S,5S,8R.

The molecular packing is stabilized by weak non-classical intermolecular C–H···O hydrogen bonds and by intermolecular C–H···π interaction between methine H atom of perhydrofurofuranyl system and the triacetoxyphenyl ring of an adjacent molecule (Table 1, *Cg1* is the centroid of C42—C47 phenyl ring).

### Experimental

3,4,5-triacetoxybenzoic acid(2.96 g, 10 mmol), Isosorbide Mononitrate (1.91 g, 10 mmol, CAS No:16051–77-7, [α]_D_=168 ° (c=1.0, EtOH) and DMAP (0.24 g, 1 mmol) were dissolved in 100 ml dry THF, then DCC (4.12 g, 10 mmol) was added to the solution at 0°C. The mixture was stirred at room temperature for 5 h. The resulting mixture was filtered and concentrated *in vacuo*. The crude product was purified by column chromatography over silica gelusing ethyl acetate/*n*-hexane (7:3) as eluent. Yield: 4.21 g (85%). Single crystals of the title compound suitable for X-ray diffraction was recrystallized from hexane/ethyl acetate (1:1).

### Refinement

All H atoms were positioned geometrically and treated as riding with aromatic C—H = 0.93 Å, methine C—H = 0.98 Å, methylene C—H = 0.97Å & methyl C—H = 0.96 Å. The H atom isotropic displacement parameters were fixed; *U*_iso_(aromatic H, methine H) = 1.2 times *U*_eq_ of the parent atom; *U*_iso_(methylene H, methyl H) = 1.5 times *U*_eq_ of the parent atom.

In the absence of significant anomalous scattering, the absolute configuration could not be reliably determined and then the Friedel pairs were merged and any references to the Flack parameter were removed. The enantiomer has been assigned by reference to unchanging chiral centres in the synthetic procedure.

### Crystal data

**Table:**

C_19_H_19_NO_13_	*F*(000) = 488
*M**_r_* = 469.35	*D*_x_ = 1.433 Mg m^−^^3^
Monoclinic, *P*2_1_	Mo *K*α radiation, λ = 0.71073 Å
Hall symbol: P 2yb	Cell parameters from 1117 reflections
*a* = 10.8053 (19) Å	θ = 2.7–19.9°
*b* = 6.5941 (12) Å	µ = 0.12 mm^−^^1^
*c* = 16.075 (3) Å	*T* = 296 K
β = 108.243 (3)°	Block, colorless
*V* = 1087.8 (3) Å^3^	0.31 × 0.25 × 0.14 mm
*Z* = 2	

### Data collection

**Table:**

Bruker APEXII CCD area-detector diffractometer	2112 independent reflections
Radiation source: fine-focus sealed tube	1394 reflections with *I* > 2σ(*I*)
graphite	*R*_int_ = 0.023
φ and ω scans	θ_max_ = 25.1°, θ_min_ = 2.0°
Absorption correction: multi-scan (*SADABS*; Bruker, 2000)	*h* = −12→12
*T*_min_ = 0.962, *T*_max_ = 0.983	*k* = −7→7
5474 measured reflections	*l* = 0→19

### Refinement

**Table:**

Refinement on *F*^2^	Primary atom site location: structure-invariant direct methods
Least-squares matrix: full	Secondary atom site location: difference Fourier map
*R*[*F*^2^ > 2σ(*F*^2^)] = 0.039	Hydrogen site location: inferred from neighbouring sites
*wR*(*F*^2^) = 0.105	H-atom parameters constrained
*S* = 1.01	*w* = 1/[σ^2^(*F*_o_^2^) + (0.0523*P*)^2^] where *P* = (*F*_o_^2^ + 2*F*_c_^2^)/3
2112 reflections	(Δ/σ)_max_ < 0.001
301 parameters	Δρ_max_ = 0.12 e Å^−^^3^
0 restraints	Δρ_min_ = −0.13 e Å^−^^3^

### Special details

**Table:**

Geometry. All esds (except the esd in the dihedral angle between two l.s. planes) are estimated using the full covariance matrix. The cell esds are taken into account individually in the estimation of esds in distances, angles and torsion angles; correlations between esds in cell parameters are only used when they are defined by crystal symmetry. An approximate (isotropic) treatment of cell esds is used for estimating esds involving l.s. planes.
Refinement. Refinement of *F*^2^ against ALL reflections. The weighted *R*-factor *wR* and goodness of fit *S* are based on *F*^2^, conventional *R*-factors *R* are based on *F*, with *F* set to zero for negative *F*^2^. The threshold expression of *F*^2^ > σ(*F*^2^) is used only for calculating *R*-factors(gt) *etc*. and is not relevant to the choice of reflections for refinement. *R*-factors based on *F*^2^ are statistically about twice as large as those based on *F*, and *R*- factors based on ALL data will be even larger.

### Fractional atomic coordinates and isotropic or equivalent isotropic displacement parameters (Å^2^)

**Table:**

	*x*	*y*	*z*	*U*_iso_*/*U*_eq_	
N1	−0.0658 (8)	0.548 (4)	0.4472 (4)	0.1120 (17)	
O2	−0.0166 (3)	0.369 (4)	0.28660 (18)	0.0708 (8)	
O6	0.0245 (3)	0.779 (4)	0.21440 (17)	0.0650 (8)	
O8	0.5258 (2)	−0.138 (4)	0.32531 (17)	0.0659 (8)	
O9	0.3795 (4)	−0.169 (4)	0.39702 (18)	0.0954 (12)	
O10	0.5277 (3)	−0.439 (4)	0.20426 (18)	0.0688 (8)	
O11	0.4726 (3)	−0.581 (4)	0.3144 (2)	0.0781 (9)	
O12	0.3201 (3)	−0.487 (4)	0.05899 (17)	0.0737 (8)	
O13	0.3616 (5)	−0.318 (4)	−0.0486 (2)	0.1167 (14)	
O41	0.1465 (2)	0.293 (4)	0.17708 (15)	0.0585 (7)	
O42	0.0434 (3)	0.151 (4)	0.04764 (18)	0.0826 (10)	
O81	−0.0735 (3)	0.671 (4)	0.37604 (19)	0.0816 (9)	
O82	0.0378 (6)	0.480 (4)	0.4869 (3)	0.158 (2)	
O83	−0.1700 (6)	0.521 (4)	0.4569 (4)	0.172 (2)	
C1	0.0854 (4)	0.513 (4)	0.3148 (2)	0.0605 (11)	
H1	0.1656	0.4496	0.3520	0.073*	
C3	−0.0643 (4)	0.367 (4)	0.1932 (3)	0.0649 (11)	
H3A	−0.1403	0.4533	0.1719	0.078*	
H3B	−0.0876	0.2303	0.1715	0.078*	
C4	0.0463 (4)	0.446 (4)	0.1642 (2)	0.0565 (10)	
H4	0.0175	0.5008	0.1046	0.068*	
C5	0.1061 (4)	0.605 (4)	0.2330 (2)	0.0548 (10)	
H5	0.1975	0.6348	0.2395	0.066*	
C7	0.0305 (6)	0.868 (4)	0.2950 (3)	0.0952 (17)	
H7A	−0.0496	0.9404	0.2899	0.114*	
H7B	0.1025	0.9630	0.3130	0.114*	
C8	0.0497 (5)	0.700 (4)	0.3605 (3)	0.0670 (11)	
H8	0.1190	0.7322	0.4149	0.080*	
C10	0.4899 (5)	−0.151 (4)	0.3995 (3)	0.0729 (13)	
C11	0.6060 (5)	−0.142 (4)	0.4792 (3)	0.112 (2)	
H11A	0.6213	−0.0044	0.4991	0.168*	
H11B	0.6808	−0.1921	0.4655	0.168*	
H11C	0.5910	−0.2243	0.5244	0.168*	
C12	0.5425 (4)	−0.574 (4)	0.2703 (3)	0.0662 (12)	
C13	0.6544 (4)	−0.711 (4)	0.2740 (3)	0.0902 (15)	
H13A	0.6682	−0.8033	0.3223	0.135*	
H13B	0.7315	−0.6313	0.2817	0.135*	
H13C	0.6353	−0.7865	0.2204	0.135*	
C14	0.3353 (5)	−0.473 (4)	−0.0211 (3)	0.0778 (14)	
C15	0.3109 (6)	−0.673 (4)	−0.0662 (3)	0.1031 (18)	
H15A	0.3250	−0.6620	−0.1221	0.155*	
H15B	0.2225	−0.7137	−0.0745	0.155*	
H15C	0.3693	−0.7721	−0.0311	0.155*	
C41	0.1323 (4)	0.151 (4)	0.1152 (2)	0.0600 (11)	
C42	0.2370 (4)	−0.002 (4)	0.1410 (2)	0.0518 (9)	
C43	0.3335 (4)	0.005 (4)	0.2218 (2)	0.0544 (10)	
H43	0.3348	0.1103	0.2605	0.065*	
C44	0.4267 (4)	−0.144 (4)	0.2443 (2)	0.0535 (10)	
C45	0.4261 (4)	−0.300 (4)	0.1875 (2)	0.0577 (10)	
C46	0.3280 (4)	−0.310 (4)	0.1084 (2)	0.0567 (10)	
C47	0.2337 (4)	−0.165 (4)	0.0849 (2)	0.0576 (10)	
H47	0.1675	−0.1745	0.0316	0.069*	

### Atomic displacement parameters (Å^2^)

**Table:**

	*U*^11^	*U*^22^	*U*^33^	*U*^12^	*U*^13^	*U*^23^
N1	0.154 (5)	0.100 (4)	0.110 (4)	0.009 (4)	0.083 (4)	0.021 (3)
O2	0.100 (2)	0.0474 (17)	0.0766 (19)	−0.0117 (17)	0.0451 (17)	0.0010 (16)
O6	0.087 (2)	0.0495 (16)	0.0616 (16)	0.0065 (16)	0.0272 (14)	0.0106 (14)
O8	0.0588 (16)	0.0684 (19)	0.0611 (16)	−0.0001 (15)	0.0055 (14)	−0.0052 (16)
O9	0.086 (2)	0.139 (3)	0.0596 (17)	0.009 (3)	0.0203 (17)	0.007 (2)
O10	0.0723 (19)	0.074 (2)	0.0657 (17)	0.0082 (17)	0.0301 (15)	0.0047 (16)
O11	0.092 (2)	0.069 (2)	0.0833 (19)	−0.0089 (18)	0.0409 (18)	−0.0031 (18)
O12	0.099 (2)	0.0669 (19)	0.0561 (16)	−0.0005 (19)	0.0261 (15)	−0.0150 (16)
O13	0.169 (4)	0.120 (4)	0.081 (2)	−0.016 (4)	0.067 (3)	−0.010 (3)
O41	0.0613 (16)	0.0570 (17)	0.0504 (15)	0.0048 (15)	0.0076 (12)	−0.0069 (14)
O42	0.097 (2)	0.077 (2)	0.0528 (15)	0.0207 (19)	−0.0076 (16)	−0.0134 (17)
O81	0.093 (2)	0.074 (2)	0.0826 (19)	0.0111 (19)	0.0350 (18)	−0.002 (2)
O82	0.174 (5)	0.188 (6)	0.125 (4)	0.050 (5)	0.064 (4)	0.085 (4)
O83	0.195 (5)	0.151 (5)	0.233 (6)	0.010 (5)	0.161 (5)	0.039 (5)
C1	0.073 (3)	0.057 (3)	0.050 (2)	0.010 (3)	0.0168 (19)	0.008 (2)
C3	0.054 (2)	0.052 (2)	0.082 (3)	−0.001 (2)	0.012 (2)	0.004 (2)
C4	0.061 (2)	0.056 (2)	0.047 (2)	0.005 (2)	0.0093 (19)	−0.0003 (19)
C5	0.062 (2)	0.051 (2)	0.051 (2)	0.001 (2)	0.0169 (19)	0.0018 (19)
C7	0.156 (5)	0.059 (3)	0.085 (3)	0.019 (4)	0.059 (3)	0.001 (3)
C8	0.083 (3)	0.060 (3)	0.060 (2)	0.005 (2)	0.026 (2)	−0.001 (2)
C10	0.078 (3)	0.078 (3)	0.055 (3)	0.004 (3)	0.009 (2)	−0.011 (2)
C11	0.091 (3)	0.160 (6)	0.063 (3)	0.019 (4)	−0.008 (3)	−0.013 (4)
C12	0.071 (3)	0.057 (3)	0.071 (3)	−0.016 (2)	0.023 (2)	−0.008 (2)
C13	0.069 (3)	0.074 (3)	0.132 (4)	0.013 (3)	0.036 (3)	0.014 (3)
C14	0.077 (3)	0.099 (4)	0.056 (3)	0.009 (3)	0.018 (2)	−0.021 (3)
C15	0.114 (4)	0.109 (5)	0.083 (3)	0.006 (4)	0.026 (3)	−0.038 (4)
C41	0.082 (3)	0.052 (3)	0.044 (2)	0.001 (2)	0.017 (2)	−0.001 (2)
C42	0.061 (2)	0.048 (2)	0.045 (2)	−0.001 (2)	0.0135 (18)	−0.0041 (19)
C43	0.066 (2)	0.047 (2)	0.049 (2)	−0.008 (2)	0.0159 (19)	−0.0077 (19)
C44	0.052 (2)	0.057 (3)	0.048 (2)	−0.005 (2)	0.0107 (18)	−0.007 (2)
C45	0.059 (2)	0.061 (3)	0.056 (2)	0.006 (2)	0.021 (2)	0.006 (2)
C46	0.072 (3)	0.054 (2)	0.046 (2)	−0.006 (2)	0.021 (2)	−0.010 (2)
C47	0.071 (3)	0.057 (3)	0.0437 (19)	−0.004 (2)	0.0153 (19)	−0.005 (2)

### Geometric parameters (Å, °)

**Table:**

O2—C1	1.42 (3)	C14—C15	1.49 (3)
O2—C3	1.427 (5)	C41—C42	1.48 (3)
O6—C5	1.42 (3)	C42—C43	1.389 (5)
O6—C7	1.405 (16)	C42—C47	1.40 (3)
O8—C10	1.367 (6)	C43—C44	1.37 (3)
O8—C44	1.404 (4)	C44—C45	1.37 (3)
O9—C10	1.187 (8)	C45—C46	1.379 (5)
O10—C12	1.36 (2)	C46—C47	1.36 (3)
O10—C45	1.39 (2)	C1—H1	0.9800
O11—C12	1.188 (6)	C3—H3A	0.9700
O12—C14	1.351 (6)	C3—H3B	0.9700
O12—C46	1.40 (3)	C4—H4	0.9800
O13—C14	1.18 (3)	C5—H5	0.9800
O41—C4	1.45 (3)	C7—H7A	0.9700
O41—C41	1.34 (3)	C7—H7B	0.9700
O42—C41	1.204 (5)	C8—H8	0.9800
O81—N1	1.38 (2)	C11—H11A	0.9600
O81—C8	1.442 (8)	C11—H11B	0.9600
O82—N1	1.189 (17)	C11—H11C	0.9600
O83—N1	1.197 (12)	C13—H13A	0.9600
C1—C5	1.527 (15)	C13—H13B	0.9600
C1—C8	1.55 (3)	C13—H13C	0.9600
C3—C4	1.505 (14)	C15—H15A	0.9600
C4—C5	1.51 (3)	C15—H15B	0.9600
C7—C8	1.50 (3)	C15—H15C	0.9600
C10—C11	1.487 (7)	C43—H43	0.9300
C12—C13	1.50 (2)	C47—H47	0.9300
			
C1—O2—C3	109.4 (13)	O12—C46—C47	121.4 (8)
C5—O6—C7	107.3 (10)	C45—C46—C47	121.0 (18)
C10—O8—C44	117.7 (4)	C42—C47—C46	119.7 (7)
C12—O10—C45	118.5 (7)	O2—C1—H1	111.00
C14—O12—C46	119 (2)	C5—C1—H1	111.00
C4—O41—C41	118.3 (7)	C8—C1—H1	111.00
N1—O81—C8	113.7 (11)	O2—C3—H3A	111.00
O81—N1—O82	117.9 (10)	O2—C3—H3B	111.00
O81—N1—O83	112.3 (12)	C4—C3—H3A	111.00
O82—N1—O83	129.8 (18)	C4—C3—H3B	111.00
O2—C1—C5	107.4 (8)	H3A—C3—H3B	109.00
O2—C1—C8	113.7 (8)	O41—C4—H4	113.00
C5—C1—C8	102.6 (18)	C3—C4—H4	113.00
O2—C3—C4	105.2 (7)	C5—C4—H4	113.00
O41—C4—C3	110.2 (19)	O6—C5—H5	114.00
O41—C4—C5	104.4 (8)	C1—C5—H5	114.00
C3—C4—C5	102.2 (8)	C4—C5—H5	114.00
O6—C5—C1	104.7 (10)	O6—C7—H7A	110.00
O6—C5—C4	108.1 (9)	O6—C7—H7B	110.00
C1—C5—C4	102.1 (17)	C8—C7—H7A	110.00
O6—C7—C8	107 (2)	C8—C7—H7B	110.00
O81—C8—C1	111.0 (18)	H7A—C7—H7B	109.00
O81—C8—C7	106.7 (12)	O81—C8—H8	111.00
C1—C8—C7	104.9 (9)	C1—C8—H8	111.00
O8—C10—O9	122.2 (4)	C7—C8—H8	111.00
O8—C10—C11	110.8 (5)	C10—C11—H11A	110.00
O9—C10—C11	126.9 (5)	C10—C11—H11B	109.00
O10—C12—O11	123.5 (18)	C10—C11—H11C	109.00
O10—C12—C13	108.9 (8)	H11A—C11—H11B	109.00
O11—C12—C13	127.6 (19)	H11A—C11—H11C	110.00
O12—C14—O13	122 (2)	H11B—C11—H11C	109.00
O12—C14—C15	110.4 (19)	C12—C13—H13A	110.00
O13—C14—C15	127.6 (8)	C12—C13—H13B	109.00
O41—C41—O42	123 (2)	C12—C13—H13C	109.00
O41—C41—C42	111.7 (8)	H13A—C13—H13B	110.00
O42—C41—C42	125 (2)	H13A—C13—H13C	109.00
C41—C42—C43	121.7 (17)	H13B—C13—H13C	110.00
C41—C42—C47	118.9 (7)	C14—C15—H15A	109.00
C43—C42—C47	119.3 (17)	C14—C15—H15B	109.00
C42—C43—C44	119.8 (18)	C14—C15—H15C	109.00
O8—C44—C43	120.5 (18)	H15A—C15—H15B	109.00
O8—C44—C45	118.8 (17)	H15A—C15—H15C	110.00
C43—C44—C45	120.7 (7)	H15B—C15—H15C	109.00
O10—C45—C44	121.7 (7)	C42—C43—H43	120.00
O10—C45—C46	118.7 (16)	C44—C43—H43	120.00
C44—C45—C46	119.4 (17)	C42—C47—H47	120.00
O12—C46—C45	117.1 (18)	C46—C47—H47	120.00
			
C3—O2—C1—C5	−2(2)	O2—C1—C8—C7	−106.1 (12)
C3—O2—C1—C8	111.2 (14)	C5—C1—C8—O81	124.5 (9)
C1—O2—C3—C4	24 (2)	C5—C1—C8—C7	9.6 (7)
C7—O6—C5—C1	38.2 (16)	O2—C3—C4—O41	75 (2)
C7—O6—C5—C4	146.4 (11)	O2—C3—C4—C5	−36 (2)
C5—O6—C7—C8	−32.0 (12)	O41—C4—C5—O6	168.9 (10)
C44—O8—C10—O9	1(4)	O41—C4—C5—C1	−81.0 (10)
C44—O8—C10—C11	−180 (2)	C3—C4—C5—O6	−76.2 (15)
C10—O8—C44—C43	63 (3)	C3—C4—C5—C1	33.9 (15)
C10—O8—C44—C45	−118 (2)	O6—C7—C8—O81	−105.3 (13)
C45—O10—C12—O11	1(3)	O6—C7—C8—C1	12.6 (7)
C45—O10—C12—C13	178.2 (15)	O41—C41—C42—C43	2(2)
C12—O10—C45—C44	70 (3)	O41—C41—C42—C47	177.7 (14)
C12—O10—C45—C46	−115.3 (16)	O42—C41—C42—C43	−177.5 (16)
C46—O12—C14—O13	4.0 (11)	O42—C41—C42—C47	−2(2)
C46—O12—C14—C15	−174.5 (6)	C41—C42—C43—C44	177.9 (14)
C14—O12—C46—C45	−118.2 (11)	C47—C42—C43—C44	2(2)
C14—O12—C46—C47	69.8 (11)	C41—C42—C47—C46	−178.7 (13)
C41—O41—C4—C3	83.5 (13)	C43—C42—C47—C46	−3(2)
C41—O41—C4—C5	−167.3 (14)	C42—C43—C44—O8	179.3 (14)
C4—O41—C41—O42	4(2)	C42—C43—C44—C45	0(2)
C4—O41—C41—C42	−175.7 (13)	O8—C44—C45—O10	−7(2)
N1—O81—C8—C1	78.0 (16)	O8—C44—C45—C46	178.8 (14)
N1—O81—C8—C7	−168.3 (17)	C43—C44—C45—O10	172.3 (15)
C8—O81—N1—O82	0(3)	C43—C44—C45—C46	−2(2)
C8—O81—N1—O83	−178 (2)	O10—C45—C46—O12	15 (2)
O2—C1—C5—O6	91.8 (18)	O10—C45—C46—C47	−173.0 (15)
O2—C1—C5—C4	−20.9 (17)	C44—C45—C46—O12	−170.4 (13)
C8—C1—C5—O6	−28.4 (11)	C44—C45—C46—C47	2(2)
C8—C1—C5—C4	−141.0 (9)	O12—C46—C47—C42	172.6 (13)
O2—C1—C8—O81	8.8 (14)	C45—C46—C47—C42	1(2)

### Hydrogen-bond geometry (Å, °)

**Table:**

*D*—H···*A*	*D*—H	H···*A*	*D*···*A*	*D*—H···*A*
C5—H5···Cg1^i^	0.98	2.56	3.365 (13)	139
C4—H4···O42^ii^	0.98	2.53	3.507 (15)	177
C43—H43···O11^i^	0.93	2.51	3.25 (3)	136
C47—H47···O42^iii^	0.93	2.52	3.314 (14)	144
C11—H11B···O83^iv^	0.96	2.52	3.39 (3)	151

Symmetry codes: (i) *x*, *y*+1, *z*; (ii) −*x*, *y*+1/2, −*z*; (iii) −*x*, *y*−1/2, −*z*; (iv) *x*+1, *y*−1, *z*.
